# Complete genome sequence of new unique recombinant HIV type 1 isolated from a child born to an HIV-infected mother

**DOI:** 10.1186/1471-2334-14-S2-P62

**Published:** 2014-05-23

**Authors:** S Sasinovich, V Eremin, E Gasich, M Thomson

**Affiliations:** 1 RRPC for Epidemiology and Microbiology, Minsk, Belorussia

## Introduction

**Figure 1:**
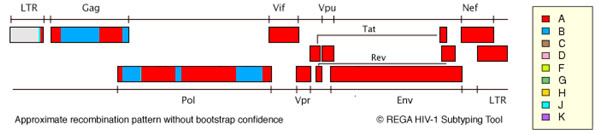


In our previously studies we detect a HIV recombinant form with BgagApolAenv by isolate named Mos. This virus differs from CRF02_AB with AgagBpolBenv. For more detailed studying of isolate Mos genome we carried out complete genome sequencing. (Figure 1)

## Materials and methods

HIV-1 RNA was extracted from plasma, reverse transcribed, and amplified using sets of primers designed to span the complete genome of HIV-1. Contig assembled in SeqMan®, DNAStar. Recombination analysis was performed using REGA HIV Subtyping Tool. Phylogenetic analysis was made in MEGA4 software with Kimura-2-parametres.

## Results

Analysis of complete genome shows that recombinant Mos is a new HIV-1 URF with gag/pol mosaic structure composed of parental subtypes A and B and rest genome composed of subtype A. Phylogenetical analysis had shown that HIV-1 subtype A ancestor is AY500393 Russia, subtype B - DQ207943 Georgia.

## Conclusions

This study confirms the presence of a unique recombinant HIV-1 strain, emerging as a result of recombination between HIV-1 strains from Russia and Georgia.

